# Cost-effectiveness of Triple Therapy with Telaprevir for Chronic Hepatitis C Virus Patients in Germany

**Published:** 2013-12-18

**Authors:** Jona T. Stahmeyer, Svenja Schauer, Siegbert Rossol, Hans Heinrich Wedemeyer, Daniel Wirth, Florence Bianic, Christian Krauth

**Affiliations:** 1 Hannover Medical School, Hannover, Germany; 2 Krankenhaus Nordwest Medical Clinic, Frankfurt, Germany; 3 Janssen-Cilag GmbH, Neuss, Germany; 4 OptumInsight, Uxbridge, United Kingdom

**Keywords:** germany, markov model, triple-therapy, cost-effectiveness analysis, hepatitis c

## Abstract

**Background:** About 400,000-500,000 people are infected with hepatitis C in Germany. Long-term consequences are the development of liver cirrhosis and hepatocellular carcinoma. The introduction of first generation protease inhibitors has significantly improved the treatment of hepatitis C genotype 1 patients. The aim of the study was to assess the cost-effectiveness of triple therapy with telaprevir in Germany.

**Methods:** We used a Markov model on disease progression and natural history to assess the cost-effectiveness of triple therapy with telaprevir compared to standard treatment with pegylated interferon and ribavirin. Model structure and inputs were discussed with clinical experts. Deterministic and probabilistic sensitivity analyses were performed to verify the robustness of results.

**Results:** The base-case analyses shows that triple therapy results in higher costs (untreated patients: €48,446 vs. €30,691; previously treated patients: €63,228 vs. €48,603) and better outcomes (untreated patients: 16.85 qualily of life years [QALYs] vs. 15.97 QALYs; previously treated patients: 14.16 QALYs vs. 12.89 QALYs). The incremental cost-effectiveness ratio (ICER) was €20,131 per QALY and €30,567 per life year gained (LYG) for previously untreated patients. ICER in treatment experienced patients was €7,664 per QALY for relapse patients, €12,506 per QALY for partial responders and €28,429 per QALY for null responders. Results were robust in sensitivity analyses.

**Conclusion:** Although triple therapy with telaprevir leads to additional costs, there is a high probability of being cost-effective for different thresholds. This health economic analysis makes an important contribution to current debates on cost savings and efficient resource allocation in the German healthcare sector.

## 1. Background

Viral hepatitis is a major public health problem affecting millions of people worldwide. Globally, about 150 million people are chronically infected with hepatitis C, of these 17.5 million people in Europe.[1,2] Data from the German Health Interview and Examination Survey (DEGS1) show an anti-HCV prevalence of 0.3% in the general population.[3] Taking risk-groups such as drug users and prison inmates who were not represented into account, about 400 to 500 thousand people are affected in Germany.[4-6] The majority of these patients have acquired genotype 1 hepatitis C virus (HCV) (61.7%).[5] In its decision on the added benefit of previously introduced first generation protease inhibitors for the treatment of genotype 1 patients, the "Gemeinsamer Bundesausschuss" (Joint Federal Committee) assumes that there are 46,000 patients with diagnosed chronic hepatitis C of genotype 1 eligible for therapy in Germany.[7,8]

Long-term organic consequences of chronic hepatitis C include the development of terminal liver cirrhosis, hepatocellular carcinoma (HCC) and premature death. Globally, 27% of liver cirrhosis and 25% of HCC are estimated to be attributable to HCV.[9] In order to prevent severe stages of liver disease and premature death, the achievement of SVR is a major outcome in the management of hepatitis C. Viral eradication significantly reduces the development of cirrhosis and related complications while increasing quality of life of infected patients.[10-14]

Genotype 1 is the most difficult to eradicate genotype and only 40-50% of patients treated with dual therapy of pegylated interferon and ribavirin (PR) achieve sustained virological response (SVR). Genotypes 2 and 3 are easier to eradicate and 80% of patients treated with PR achieve SVR.[15,16] Current German and European guidelines for the treatment of chronic hepatitis C were updated in 2010 and 2011, respectively. They provide recommendations on diagnostic procedures for the initial evaluation, choice of therapy and management of complications of hepatitis C.[17,18] Guidelines recommend dual antiviral therapy with pegylated interferon and ribavirin. The introduction of first generation protease inhibitors (telaprevir and boceprevir) for the more difficult to treat genotype 1 patients in 2011 dramatically improved treatment SVR rates for naïve as well as treatment experienced patients while also shortening treatment duration for many patients.[19-22] Triple therapy with pegylated interferon, ribavirin and a protease inhibitor is now established as the new standard of care for HCV genotype 1 patients.[6,23,24] Treatment experienced patients who failed previous treatment attempts with dual therapy especially benefit from these new treatment options, since past retreatment with dual therapy was associated with poor response rates. Although these newly introduced treatment options lead to a significant increase of SVR rates, they induce additional costs and cause more adverse events.[25-27]

Recent studies by Camma *et al.* and Liu *et al.* have estimated the costs-effectiveness of previously introduced first generation protease inhibitors for treatment naïve patients in Italy and the United States. Curtis *et al.* estimated the cost effectiveness of for both treatment naïve and treatment experienced patients in the United Kingdom.[28-30] Camma *et al.* analysed the effectiveness of five different protease inhibitor treatment strategies compared to dual therapy. They state that triple therapy is highly cost-effective.[28] Liu *et al.* conclude for a US setting that triple therapy offers new effective opportunities in treating HCV patients, even though additional benefits come along with increased adverse effects and notably higher costs.[29] Curtis *et al.* analyzed cost effectiveness of telaprevir triple therapy in a UK setting and conclude that telaprevir triple therapy is highly cost effective in that setting.[30] Based on this model the National Institute for Clinical Excellence (NICE) in its technology appraisal conclude that telaprevir triple therapy "represents a cost-effective use of NHS resources and should be recommended as an option for the treatment of genotype 1 chronic hepatitis C in adults with compensated liver disease who are previously untreated or in whom previous treatment has failed".[31]

Transferability of the results of these analyses to the setting of the German healthcare system is limited, for they have been created from very different healthcare and cost settings in the respective countries mentioned. Moreover, there are several methodological differences between these studies, e.g. time horizon, perspective and patient characteristics.[32] The aim of our study was to perform a cost-effectiveness analysis of previously introduced triple therapy with telaprevir for both treatment naïve and treatment experienced patients in comparison to dual therapy, considering the German healthcare system. The analysis is performed taking into account the statutory health insurance perspective.

## 2. Materials and Methods

We used a previously published Markov model of HCV natural history and disease progression for the United Kingdom.[30] Cycle length was 1 year, horizon is lifetime and half-cycle correction was applied. The model assesses the cost-effectiveness of triple-therapy with telaprevir for treatment-naïve and treatment-experienced patients with a chronic genotype 1 HCV infection in Germany. Patient cohorts are defined by age and grouped by severity of disease. Severity of disease was classified by initial fibrosis stage based on Metavir score (mild HCV: F0, F1; moderate HCV: F2, bridging fibrosis/cirrhosis: F3, F4). Treatment naïve patients were stratified in three age groups starting at an age of 30, 40 or 50 years and severity of disease based on the Action in Diabetes and Vascular Disease: Preterax and Diamicron MR Controlled Evaluation (ADVANCE) study.[19] The starting age of treatment for all experienced patients (relapsers, partial and null responders) was defined as 50 years, taking into account the patient age distribution and median patient age from REALIZE study data.[20]

### *Natural History Model*

The natural history model presented in Figure 1 simulates the lifetime progression of patients with chronic HCV infection. Progression is characterized by stages of disease severity and transition to a worse health state can occur on a yearly basis. At earlier stages, disease progression rates are age dependent. Health states include mild HCV, moderate HCV, compensated cirrhosis, decompensated cirrhosis, HCC and liver transplantation/post-liver transplantation. Costs and utilities are associated with each health state. Transition probabilities were adapted from the study by Curtis *et al.*, which used data from previously published studies.[33,34] Patients achieving SVR will transition to recovered health states, depending on prior severity of disease. Disease-specific death can occur from decompensated cirrhosis, HCC, liver transplantation/post liver transplantation. Background mortality was included for all patients based on national mortality rates.[35] A compilation of model schematics and patient group characteristics is presented in Figure 1.

We performed a systematic literature search in PubMed to determine adequate, up-to-date information on hepatitis C for model inputs. In comparison to the original model by Curtis *et al.*, we adjusted several input parameter with data for the German healthcare system. These include costs for antiviral treatment (pharmaceuticals, patient monitoring, adverse events [AEs]), and different health and quality of life states.

Treatment strategies and patient characteristics were adapted from the ADVANCE and REALIZE phase-3 trials.[19,20] Patients with mild or moderate HCV or compensated cirrhosis received initial treatment at the outset of the model.

**Figure 1. Model Schematics**

**characteristics of the treatment naive patient cohort**

| disease severity | ≤ 35 years | 36-45 years | > 45 years |
|---|---|---|---|
| Mild HCV | 63.6% | 43.4% | 30.8% |
| Moderate HCV | 32.7% | 40.6% | 43.3% |
| Bridging fibrosis/cirrhosis | 3.7% | 16.0% | 25.9% |
| Age Group distribution | 15.2% | 24.2% | 60.6% |

**characteristics of the treatment experienced patient cohort**

| disease severity | relapser | partial responder | null responder |
|---|---|---|---|
| mild HCV | 25.4% | 22.4% | 13.8% |
| moderate HCV | 30.5% | 31.6% | 29.4% |
| bridging fibrosis/cirrhosis | 44.1% | 46.0% | 56.8% |
| cohort weight | 53.5% | 19.1% | 27.4% |

**Model structure**

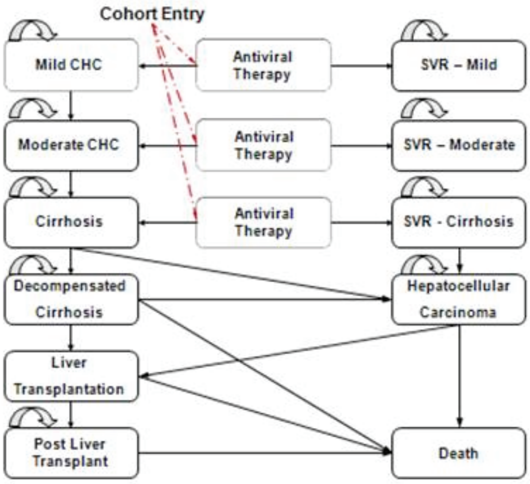

HCV=hepatitis C virus; CHC=chronic hepatitis C; SVR=sustained viral response

Triple therapy of naïve patients consists of a response-guided treatment with telaprevir in combination with PR (T/PR) for 12 weeks, followed by another 12 weeks of PR in cases of extended rapid virological response (eRVR), as defined by the summary of main product characteristics (SmPC) (virus undetectable at treatment weeks 4 and 12 of), or else 36 weeks for a total of 24 or 48 weeks, respectively. Dual therapy for naïve patients consists of treatment with PR for 48 weeks. To prevent the continuation of treatment for patients without adequate response, stopping rules were implemented in underlying trials.[19,20]

Treatment of experienced patients depended on prior response. Relapse patients received response-guided T/PR treatment in the same way as naïve patients. Partial and null responders received 12 weeks of T/PR treatment and another 36 weeks of PR alone in the triple therapy scheme. All patients in the control group received 48 weeks of PR in the dual therapy scheme with no options for reduced treatment time.[20] SVR rates were adapted from the SmPC based on calculations performed by the European Medicines Agency (EMA).[36]

The management of AEs was also included in the model. As AEs are common in the treatment of HCV infections, procedures were discussed with clinical experts. We included only severe AEs in the analysis as these have potential impact on healthcare costs. We included all of the most common AEs related to hepatitis C treatment such as rash, pruritus, nausea, diarrhea and anemia. Incidence data on side effects were extracted from the clinical study reports.[37,38] AEs were classified on the basis on the Common Terminology Criteria of Adverse Events (CTCAE), Version 3. Costs for treatment of AEs were calculated using drug costs in 2013 Euros or using costs for inpatient care in cases of patients with anemia.

*Cost Calculation*

The cost analysis was conducted for both treatment strategies, calculating costs in 2013 Euros. Costs for the treatment were calculated for drug, monitoring during treatment and potential AEs. Drug costs for PR and telaprevir were derived from the German drug directory on 01.03.2013 and adjusted according to the analysis perspective. Therefore, the results of the price negotiation on telaprevir between the manufacturer and the German head association of sickness funds were taken into account. Costs of peginterferon alpha-2a account for €242.31/week, ribavirin for €183.45/week and telaprevir for €2,576.36/week. A 48-week treatment with PR amounts for a total of €20,436. Triple therapy with T/PR results in €41,135 for a 24-week treatment and €51,353 for a 48-week treatment. Costs for therapy monitoring were based on current German and European guidelines including patient visits, diagnostic and laboratory testing and procedures.[17,18] Data were adapted from a previous study which evaluated costs of antiviral therapy and patient monitoring depending on treatment duration in context of the German healthcare system.[39] Basic diagnostic procedures amount for €302.75 for treatment naïve and €217.85 for treatment experienced patients. Monitoring costs depend on length of therapy and respective point of possible treatment discontinuation and ranges between €414.31 and €1,000.02.[39] Annual costs of different health states in subsequent years following antiviral treatment were adapted from a previous German study by Wasem *et al.* and updated to 2013 Euros.[40]

*Utilities*

Quality of life data in patients with chronic HCV infection is based on the EQ-5D and was derived from a previous study conducted by Siebert *et al.*,[41] quantifying the well-being of a patient on a scale with 0 as death and 1 being in perfect health. Reduction of quality of life during antiviral therapy was evaluated in the ADVANCE study for treatment naïve patients and in the REALIZE study for treatment experienced patients. Utility decrements were -0.114 in the year of treatment for treatment naïve patients receiving triple therapy (T/PR) and -0.123 for treatment naïve patients receiving dual therapy (PR). For all treatment experienced patient subgroups, decrement of utility was -0.163 for patients receiving triple therapy and -0.140 for patients receiving dual therapy.[30] For patients achieving SVR, an improvement of 0.05 in patients with mild chronic hepatitis C (CHC) and 0.06 for moderate CHC and compensated cirrhosis was assumed.[34]

*Further Model Inputs*

The model was constructed with Microsoft Excel 2010. We performed deterministic (DSA) and probabilistic sensitivity analyses (PSA) for major variables to examine the effect of uncertainty on primary outcomes. For variation of SVR rates and quality of life, 95% confidence intervals were used. Other parameters were varied either by 25% (transition probabilities and telaprevir costs) or 50% (health state costs), as no detailed data was available. Distributions used in PSA were derived from a recently published empirical study.[34] distributions were applied to transition probabilities and utilities and Gamma distributions were applied to costs and decrement of utilities. For SVR rates posterior distributions based on a meta-analysis were used. We followed the recommendations of the German Institute for Quality and efficiency in Health Care (IQWiG) by discounting future costs and benefits at 3% annually.[42]

## Table 1. Model Inputs

| Parameter | | Base-case (range) | Reference |
|---|---|---|---|
| **Transition Probability for Disease Progression** | | | |
| From | To | | |
| Mild HCV | Moderate HCV | | 33 |
| | ≤35 yrs | 0.015 (0.011-0.019) | |
| | 36-45 yrs | 0.023 (0.017-0.029) | |
| | >45 yrs | 0.035 (0.026-0.044) | |
| Moderate HCV | Compensated Cirrhosis | | 33 |
| | ≤35 yrs | 0.021 (0,016-0,026) | |
| | 36-45 yrs | 0.032 (0.024-0.040) | |
| | >45 yrs | 0.048 (0.036-0.060) | |
| Compensated Cirrhosis | Decompensated Cirrhosis | 0.039 (0.029-0.049) | 34 |
| | HCC | 0.014 (0.011-0.018) | |
| Compensated Cirrhosis (SVR) | Decompensated Cirrhosis | 0.000 | 34 |
| | HCC | 0.014 (0.011-0.018) | |
| Decompensated Cirrhosis | HCC | 0.014 (0.011-0.018) | 34 |
| | Liver Transplant | 0.020 (0.015-0.025) | |
| | Death | 0.130 (0.098-0.163) | |
| HCC | Liver Transplant | 0.040 (0.030-0.050) | Expert Opinion |
| | Death | 0.430 (0.323-0.538) | 34 |
| Liver transplant | Death | 0.210 (0.158-0.263) | 34 |
| Post-liver transplant | Death | 0.057 (0.043-0.071) | 34 |
| **Utilities** | | | |
| Early Stages | Mild HCV | 0.83 (0.77-0.88) | 41 |
| | Moderate HCV | 0.76 (069-0.83) | |
| | Compensated Cirrhosis | 0.74 (0.67-0.78) | |
| SVR after Treatment | Mild HCV | 0.88 (0.820.93) | 34, 41 |
| | Moderate HCV | 0.82 (0.75-0.89) | |
| | Cirrhosis | 0.80 (0.73-0.84) | |
| Advanced Stages | Decompensated Cirrhosis | 0.72 (0.66-0.79) | 41 |
| | HCC | 0.72 (0.66-0.79) | |
| | Liver Transplantation | 0.72 (0.66-0.79) | |
| | Post liver transplantation | 0.79 (0.66-0.79) | |
| Decrement Utility during Treatment | Naïve Patients T/PR | -11.4% (-5.7%/-17.1%) | |
| | Naïve Patients PR | -12.3% (-6.2%/-18.5%) | |
| | Experienced Patients T/PR | -16.3% (-12.2%/-20.4%) | |
| | Experienced Patients PR | -14.0% (-10.5%/-17.5%) | |
| **SVR Rates** | | | 43 |
| Treatment Naïve | PR | 46.0% (41.0%-51.0%) | |
| | T/PR | 78.5% (74.0%-83.0%) | |
| Relapser | PR | 24.1% (13.0%-34.0%) | |
| | T/PR | 84.1% (77.0%-90.0%) | |
| Partial Responder | PR | 14.8% (4.0%-34.0%) | |
| | T/PR | 61.2% (46.0%-75.0%) | |
| Null Responder | PR | 5.4% (1.0%-18.0%) | |
| | T/PR | 30.1% (20.0%-43.0%) | |
| **Adverse Events (>Grade 3)** | **PR** | **T/PR** | 37 |
| Rash | 0, 3% | 3, 6% | |
| Pruritus | 0, 5% | 0, 5% | |
| Nausea | 0, 5% | 1, 4% | |
| Diarrhea | 0, 5% | 0, 5% | |
| Anemia | 1, 9% | 7, 2% | |
| **Discount Rate** | | 3% (0%/6%) | 42 |
| **Weekly Drug Costs (in €)** | | | 36 |
| PEG 2a | | 242.31 | |
| Ribavirin | | 183.45 | |
| Telaprevir | | 2,576.36 (1,932.27-3,220.44) | |
| **Health States Costs (in €)** | | | 40 |
| Mild HCV | | 149.85 (74.92-224.77) | |
| Moderate HCV | | 153.03 (76.51-229.54) | |
| Compensated Cirrhosis | | 757.87 (378.94-1,136.81) | |
| Decompensated Cirrhosis | | 9,544.53 (4,772.27-14,316.80) | |
| HCC | | 23,546.05 (11,773.02-35,319.07) | |
| Liver Transplantation | | 140,204.16 (70,102.08-210,306.24) | |
| Follow-up Phase Post-liver Transplantation | | 20,277.09 (10,138.55-30,415.64) | |
| **Treatment Period Adverse Event Costs (in €)** | | | Expert opinion |
| Rash | | 26.55 | |
| Pruritus | | 22.33 | |
| Nausea | | 64.38 | |
| Diarrhea | | 2.05 | |
| Anemia | | 1,801.14 | |
| **Monitoring Costs during Treatment (in €)** | | | 39 |
| Basic Diagnostics (naïve) | | 302.75 | |
| Basic Diagnostics (experienced) | | 217.85 | |
| 4 weeks | | 414.31 | |
| 12 weeks | | 566.09 | |
| 24 weeks | | 768.76 | |
| 48 weeks | | 1,000,02 | |

HCV=hepatits C virus; SCR=sustained viral response; HCC=hepatocellular carcinoma; T=telaprevir; PR=pegylated interferon and ribavarin

Beta distributions were applied to transition probabilities and utilities and Gamma distributions were applied to costs and decrement of utilities. For SVR rates, posterior distributions based on a meta-analysis were used. We followed the recommendations of the German Institute for Quality and efficiency in Health Care (IQWiG) by discounting future costs and benefits at 3% annually.[42]

## 3. Results

### Base-case Analysis

Dual therapy with PR in treatment naïve patients resulted in total lifetime costs of €30,691 (€15,444 drug costs) and 15.97 QALYs per average treated patient. Triple therapy with T/PR resulted in total lifetime costs of €48,446 (€39,238 drug costs) and 16.85 QALYs per patient.

The likelihood of achieving SVR was 46.0% for naïve patients receiving dual therapy and 78.5% for the triple therapy (T/PR) group. Our estimates lead to an incremental cost effectiveness ratio (ICER) of €20,131/QALY and €30,567/LYG. Furthermore, triple therapy can prevent additional 184 cases of liver cirrhosis and twelve liver transplants per 1,000 patients compared to dual therapy.

The analysis of treatment experienced patients (all subgroups) receiving T/PR treatment result in total lifetime costs of €63,288 (€45,220 drug costs) and 14.16 QALYs per average treated patient. Treating patients with PR resulted in average lifetime costs of €48,603 (€20,471 drug costs) and 12.89 QALYs. This leads to an ICER of €12,321/QALY and €15,852/LYG. Additionally, triple therapy can prevent the development of 258 liver cirrhosis cases and 19 liver transplants per 1,000 patients, compared to dual therapy.

The pooled likelihood of treatment experienced patients achieving SVR was 64.9% in the T/PR group compared to 16.1% in the PR group. Patients with a prior relapse to dual therapy achieved 84.1% SVR in average under T/PR treatment and 24.1% for retreatment with PR. Partial and null responders achieved 61.2% and 30.1% SVR, respectively, under triple therapy, whereas dual therapy lead to significantly lower average response rates of 14.8% and 5.4%, respectively. Treatment of prior relapse patients had the best ICER, resulting in €7,664/QALY. Treatment of partial responders had an ICER of €12,506/QALY. Treating null responders had an ICER of €28,429/QALY. Table 2 summarizes the results of the base-case analysis.

**Table 2. Base-case Analysis Results**

| | | Costs | | Outcomes | | |
|---|---|---|---|---|---|---|
| | | Total Costs | Drug Costs | QALY | LYG | SVR |
| Treatment Naïve | Dual therapy (PR) | € 30,691 | € 15,444 | 15.97 | 19.97 | 46.00% |
| | Triple therapy (T/PR) | € 48,446 | € 39,238 | 16.85 | 20.55 | 78.50% |
| | ICER | - | - | €20,131/QALY | €30,567/LYG | - |
| Treatment Experienced (pooled) | Dual therapy (PR) | € 48,603 | € 20,471 | 12.89 | 16.86 | 16.10% |
| | Triple therapy (T/PR) | € 63,288 | € 45,220 | 14.16 | 17.85 | 64.90% |
| | ICER | - | - | €11,557/QALY | €14,868/LYG | - |
| Relapser | Dual therapy (PR) | € 45,989 | € 20,471 | 13.24 | 17.16 | 24.10% |
| | Triple therapy (T/PR) | € 58,240 | € 45,220 | 14.84 | 18.38 | 84.10% |
| | ICER | - | - | €7,664/QALY | €10,044/LYG | - |
| Partial Responder | Dual therapy (PR) | € 48,248 | € 20,471 | 12.93 | 16.92 | 14.80% |
| | Triple therapy (T/PR) | € 63,379 | € 45,220 | 14.14 | 17.86 | 61.20% |
| | ICER | - | - | €12,506/QALY | €16,046/LYG | - |
| Null Responder | Dual therapy (PR) | € 53,956 | € 20,471 | 12.2 | 16.23 | 5.40% |
| | Triple therapy (T/PR) | € 73,083 | € 45,220 | 12.87 | 16.8 | 30.10% |
| | ICER | - | - | €28,429/QALY | €33,805/LYG | - |

QALY=quality adjusted life year; LYG=life years gained; PR=pegylated interferon and ribavirin; t=telaprevir; ICER=incremental cost effectiveness ratio

*One-way Sensitivity Analyses*

We performed one-way sensitivity analyses for major model inputs including transition probabilities, utilities, drug costs, health state costs and discount rates. The most important results are presented in tornado-diagrams showing the top ten parameters with the greatest impact on costs per QALY ratio for treatment naïve and treatment experienced patients (Figure 2).

Cost-effectiveness in treatment naïve patients is highly sensitive on changes in discount rates, telaprevir costs, utility values and SVR rates. A discount rate of 0% results in an ICER of €6,621/QALY and 6% in €33,220/QALY. A reduction of telaprevir costs results in an ICER of €12,292/QALY, whereas an increase leads up to €27,969/QALY.

For example, a variation of utility using the 95% confidence interval for mild or moderate CHC patients achieving SVR showed that ICER for QALYs ranges from €17,539 to €24,471 and €16,646 to €25,460, respectively. Using upper and lower limits of the 95% confidence interval for SVR rates in triple and dual therapy, the ICER ranges from €16,879 to €24,418 and €16,565 to €24,980, respectively. In addition, impact of variables on costs per LYG was analysed. Summarizing, ICER on costs per LYG is most significantly influenced by discount rates, costs of telaprevir and SVR-rates in triple and dual therapy.

**Figure 2. Tornado Chart**

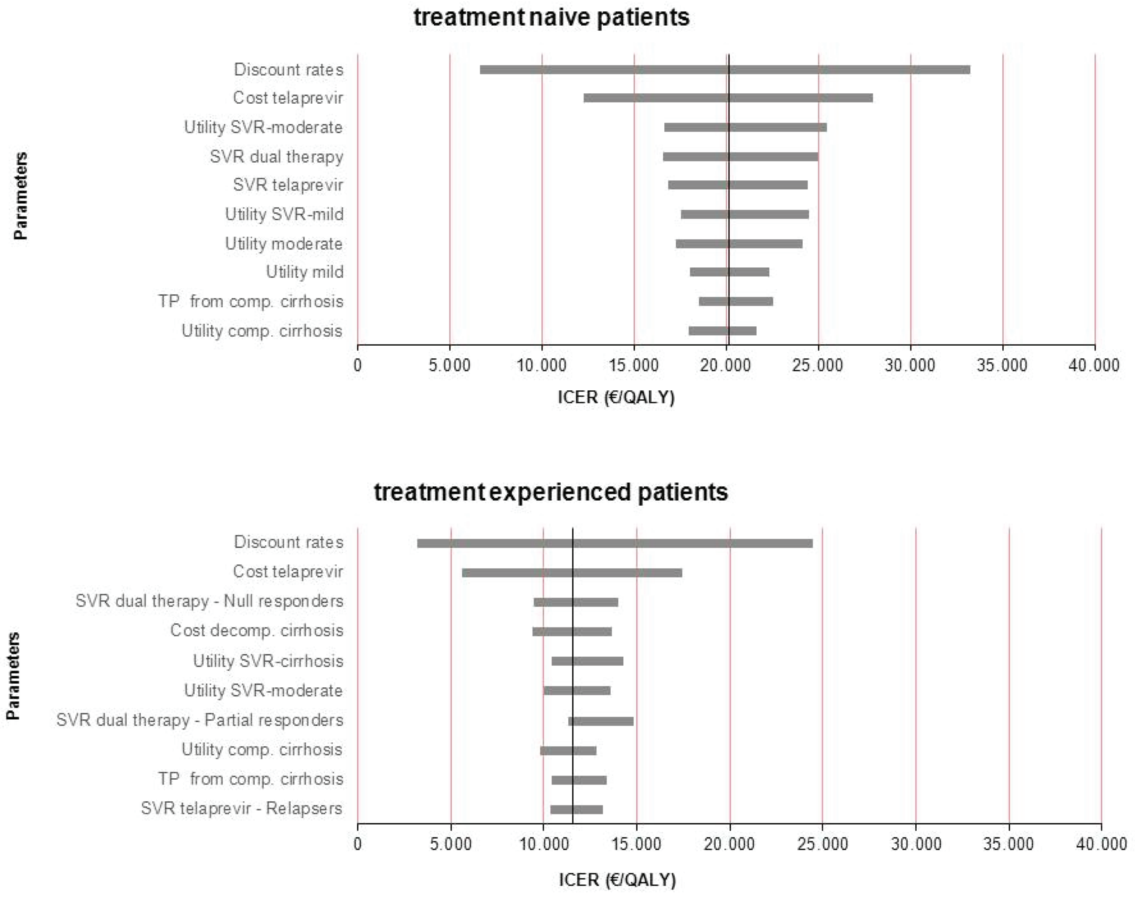

SVR=sustained viral response; ICER=incremental cost effectiveness ratio

Cost-effectiveness in treatment experienced patients is sensitive to changes of discount rates, telaprevir costs and SVR rates, whereas the impact is not as great as in the analyses in treatment naive patients. A discount rate of 0% results in an ICER of €3,206/QALY and 6% in €24.451/QALY. Reducing telaprevir costs by 25% resulted in an ICER of €5,649/QALY and an increase by 25% resulted in €17,466/QALY. A variation of ±25% in utility value of cirrhosis patients achieving SVR results in an ICER for QALYs ranging from €10,420 to €14,286. Variation of other utility values such as utility for moderate CHC patients achieving SVR or utility of patients with compensated cirrhosis achieving SVR have a lower impact on costs per QALY ratio. Additional analyses were performed for incremental costs per LYG. The cost-effectiveness ratio is most vulnerable to changes in discount rates, costs of telaprevir and SVR rates in different patient groups. Additionally transition probability from compensated to decompensated cirrhosis and costs for treating decompensated cirrhosis have a major impact on costs per LYG ratio.

*Probabilistic Sensitivity Analyses*

We used a Monte Carlo simulation for varying all variables simultaneously and executed a total of 1,000 iterations. The results of our simulation are shown in cost-effectiveness planes for treatment naïve and treatment experienced patients in Figure 3. For both treatment groups, triple therapy results in better outcomes, but also implies higher costs. The chance of being more effective and less expensive than dual therapy was 0%.

**Figure 3. Cost-effectiveness Planes**

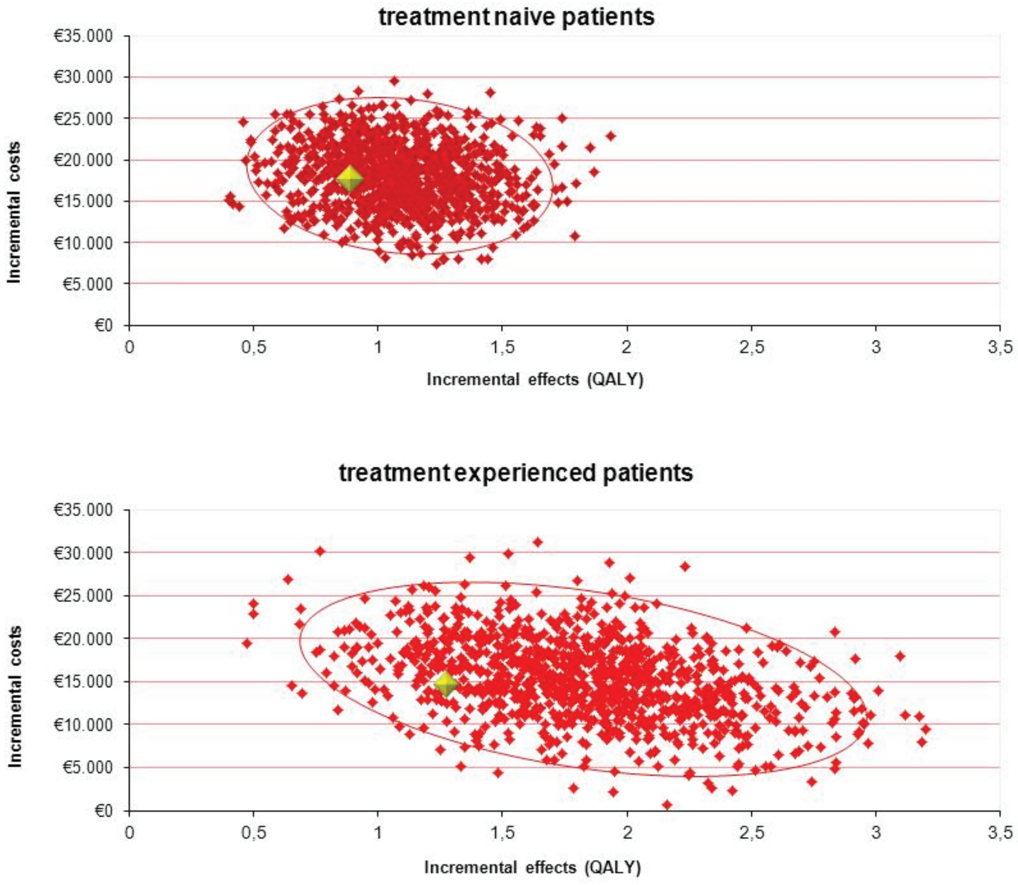

QALY=quality adjusted life year

Although there are no explicit or implicit thresholds for valuing different interventions in Germany, we evaluated the probability of being cost-effective for different QALY thresholds.

Assuming a threshold of €20,000/QALY, the probability of triple therapy being cost-effective compared to dual therapy is 69.9% for treatment naïve patients and 96.5% for treatment experienced patients. At a QALY-threshold of €30,000/QALY, the probability of being cost-effective is 94.9% for treatment naïve patients and 99.3% for treatment experienced patients. We additionally adapted QALY-thresholds of the British National Institute for Health and Clinical Excellence (NICE) of £20,000/QALY (appr. €23,000/ QALY) and £30,000/QALY (appr. €34,500/QALY). The probability of being cost-effective is 81.3%, respectively, 97.8% in treatment naïve patients and 98.5% and 99.5% for treatment experienced patients, respectively. Cost acceptability curves are presented in Figure 4.

**Figure 4. Cost-effectiveness Acceptability Curve**

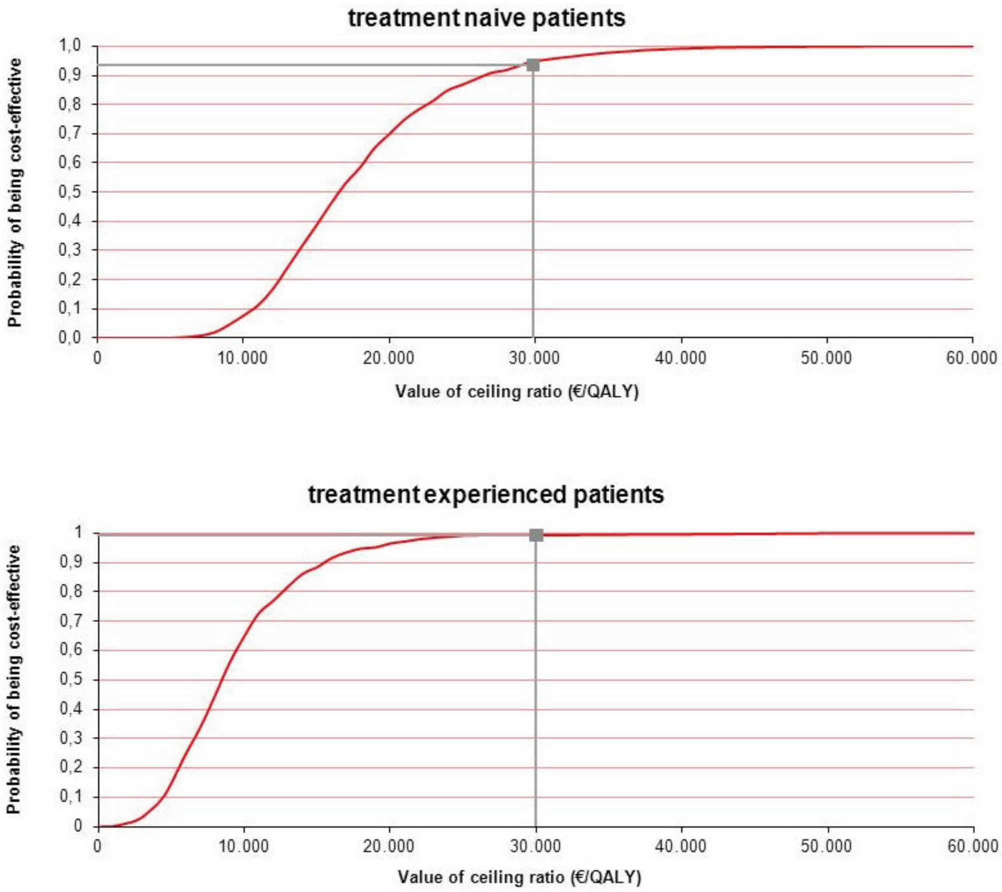

QALY = quality adjusted life year

## 4. Discussion

Our study evaluates the long-term cost-effectiveness of triple therapy with telaprevir for chronic HCV genotype 1 patients from the perspective of the German healthcare system. For both treatment naïve and treatment experienced patients, triple therapy leads to improved results in terms of SVR, QALYs and LYG when compared to dual therapy, but is more costly: Pharmaceutical costs of a 24-week triple therapy amount to €41,135, a 48-week treatment for €51,353, whereas a 48-week dual therapy results in total costs of €20,436. Clinical trials show that SVR, which is the primary goal in the therapy of chronically infected HCV patients, is increased significantly by adding telaprevir to PR.

Our model shows that these improved SVR rates for difficult to treat genotype 1 patients translate into long-term outcomes beneficial for patients. Telaprevir triple therapy is projected to help avoid the occurrence of 184 (naïve patients) and 258 (experienced patients) additional cases of liver cirrhosis, as well as 12 liver transplants in naïve patients and 19 transplants in experienced patients, per 1,000 patients.

Our calculations result in an average ICER of €20,131/QALY in treatment naïve patients. Cost-effectiveness ratios were €7,664/QALY in relapse patients, €12,506/QALY in partial responders and €28,429/QALY in null responders. The robustness of the results were tested and confirmed in multiple sensitivity analyses. Of those variables tested, discount rates, telaprevir costs and utility values had a major influence on the results. There is a high probability of triple therapy being cost-effective at different threshold levels.

We compared our results to previously published studies by Camma and colleagues and Liu and colleagues, although comparability is limited due to methodological differences between these studies (e.g. model structure, time horizon, perspective, patient characteristics). The study by Camma *et al.* determined cost-effectiveness for both protease inhibitors telaprevir and boceprevir for a 20-year time horizon using LYG and QALYs as outcomes. The telaprevir response-guided therapy resulted in €19,204/LYG and €10,755/QALY. Confirming our study results, Camma *et al.* also state that ICER for LYG is sensitive to changes in SVR rates, drug costs and transition probabilities. Due to a limited time horizon, changes in discount rates have only a minor impact on the results. Camma *et al.* additionally analyzed cost-effectiveness of a IL28B genotype-guided treatment strategy, which results in an even better cost-effectiveness ratio.

Liu *et al.* created a boceprevir and a telaprevir treatment scenario for treatment naïve patients with mild and moderate fibrosis and estimated the cost-effectiveness for the United States. They conclude that triple therapy results in better cost-effectiveness ratios for patients with advanced fibrosis when compared to patients with mild fibrosis (boceprevir scenario – mild fibrosis: $70,100/QALY, advanced fibrosis: $36,300/QALY; telaprevir scenario – mild fibrosis: $91,000/QALY, advanced fibrosis: $47,400/QALY). The results are comparable to our own study, in which treatment results in a better ICER for patients in advanced disease stages, with an ICER of €33,008/QALY for mild HCV patients, and €16,596/QALY, and €12,205/QALY for patients with moderate HCV infection and patients with bridging fibrosis/cirrhosis, respectively.

There are some limitations that have to be taken into account when interpreting the results of our study. Our model is based on efficacy data and patient characteristics from clinical Phase 3 trials. In fact, data from clinical trials are usually not easily transferable to clinical practice. Real-life populations may differ from the populations in clinical trials. Real-world efficacy data on telaprevir triple therapy are limited.[44,45] Still, those data show that a comparable number of patients achieve virological response at week 12 under triple therapy as in clinical trial settings. Unfortunately, published data lack comparison of AE rates >Grade 3 during treatment, so there is yet no insight into this aspect of treatment to compare to our study.

AEs play a major role in treating patients in clinical practice. In patients receiving triple therapy, AEs are more frequent and more severe compared to dual therapy. First real-life data on triple therapy safety in patients with advanced liver disease show a high rate of patients with anemia. Triple therapy is complex and patients need to be monitored closely by treating physicians.[24,46,47] The impact of AEs during treatment on quality of life may be underestimated in the model. However, we assume a more mature version of our model fed with additional data from real-life treatment setting, would achieve comparable results.

Additionally, control group treatment did not fully comply with current German treatment guidelines. These recommend either a 24-week, 48-week or 72-week treatment with PR, depending on viral load and response. In the ADVANCE and REALIZE trials, control patients were treated for 48 weeks, irrespective of viral load or response. However, the majority of genotype 1 patients receiving dual therapy in routine care are treated for 48 weeks,[19] which is the official recommendation in the SmPC for peginterferon alfa-2a.

A general problem in health economic modeling is the timeliness and reliability of data used. Data on pharmaceutical prices are easily and promptly available. Conversely, data such as health state costs or quality of life estimates have to be either generated or adapted and updated from previously published studies. We performed extensive literature analyses to determine the best available data for the German healthcare system. Costs in different health states and quality of life data were adapted and updated from studies performed by the German Hepatitis Model Group (GEHMO). Costs for patient monitoring during therapy is based on own estimates.[39] Model structure and input parameters were additionally discussed with clinical experts and checked for their validity. Nevertheless, there is need for more economic research in the field of hepatitis C to gather current data.

Furthermore, our model does not take into account all aspects of chronic hepatitis C and its treatment. For example, we did not examine all potential long-term damaging outcomes of hepatitis C. We also do not take into account transmissions of HCV by infected and untreated patients.

Previously introduced first generation protease inhibitors have further improved the treatment of patients with chronic genotype 1 HCV infection. Our study estimates the cost-effectiveness of triple therapy with telaprevir and therefore makes an important contribution to current debates on cost savings and efficient allocation of resources in the German healthcare sector. This study using a decision-analytic Markov model estimates that improved SVR rates observed for telaprevir triple therapy may lead to increased survival rates and quality of life. Furthermore, it may help to avoid the occurrence of additional cases of liver cirrhosis and liver transplants compared with PR treatment alone. Telaprevir triple therapy has shown to be cost effective for all patient subgroups according to label with genotype 1 HCV when adopting NICE cost thresholds to a German perspective. This analysis can serve as a basis for the health economic analysis of future treatment strategies for patients with chronic hepatitis C, which will be implemented in the beginning of 2014.

**Conflict of Interest Declaration**

The study was funded by Janssen-Cilag GmbH, Neuss, Germany. Jona T. Stahmeyer, Svenja Schauer and Christian Krauth received funding for this study. Jona T. Stahmeyer received travel grants from Janssen Cilag GmbH and Roche AG. Siegbert Rossol and Hans Heinrich Wedemeyer have done advisory work for different pharmaceutical companies and have received numerous research grants independently of the present study. Daniel Wirth is employee of Janssen-Cilag GmbH, Neuss, Germany and Florence Bianic is employee of OptumInsight, Uxbridge, UK. The authors confirm that the publication of study results was not contingent on the sponsor's approval.
